# Genetically modified T cells in cancer therapy: opportunities and challenges

**DOI:** 10.1242/dmm.018036

**Published:** 2015-04

**Authors:** Michaela Sharpe, Natalie Mount

**Affiliations:** Cell Therapy Catapult, 12th Floor Tower Wing, Guy’s Hospital, Great Maze Pond, London, SE1 9RT, UK.

**Keywords:** Immunotherapies, Gene modification, TCR, CAR, T cell, Oncology, Efficacy, Safety, Regulation, Manufacturing, Clinical trial

## Abstract

Tumours use many strategies to evade the host immune response, including downregulation or weak immunogenicity of target antigens and creation of an immune-suppressive tumour environment. T cells play a key role in cell-mediated immunity and, recently, strategies to genetically modify T cells either through altering the specificity of the T cell receptor (TCR) or through introducing antibody-like recognition in chimeric antigen receptors (CARs) have made substantial advances. The potential of these approaches has been demonstrated in particular by the successful use of genetically modified T cells to treat B cell haematological malignancies in clinical trials. This clinical success is reflected in the growing number of strategic partnerships in this area that have attracted a high level of investment and involve large pharmaceutical organisations. Although our understanding of the factors that influence the safety and efficacy of these therapies has increased, challenges for bringing genetically modified T-cell immunotherapy to many patients with different tumour types remain. These challenges range from the selection of antigen targets and dealing with regulatory and safety issues to successfully navigating the routes to commercial development. However, the encouraging clinical data, the progress in the scientific understanding of tumour immunology and the improvements in the manufacture of cell products are all advancing the clinical translation of these important cellular immunotherapies.

## Introduction

**Fig. 1.**
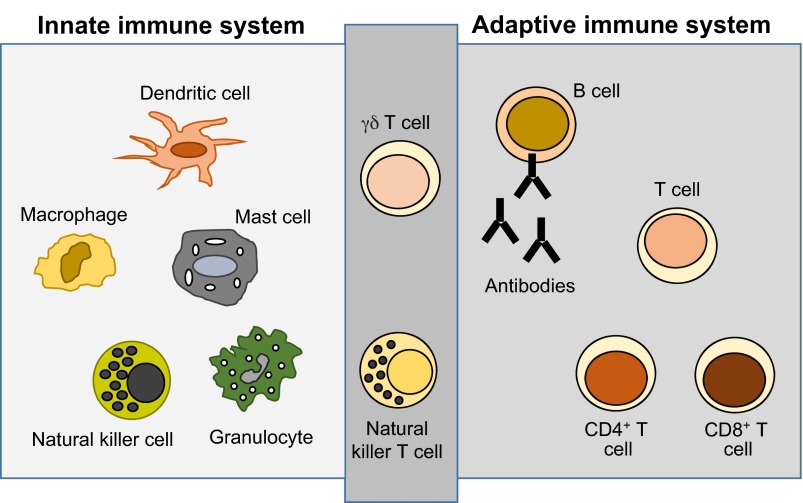
**Cells of the innate and adaptive immune systems.** The innate immune system provides an immediate response to foreign targets, with responses typically within minutes to hours. It consists of a number of soluble factors and proteins as well as a diverse set of cells, including granulocytes, macrophages, dendritic cells and natural killer cells. The second branch of the immune system is the adaptive or acquired immune system, which provides specific, long-lasting immune responses. The adaptive and innate immune systems are linked; for example dendritic cells are important adaptive immune system cell activators. The adaptive immune system consists of antibodies, B cells, and CD4^+^ and CD8^+^ T cells, and these enable a highly specific response against a particular target. Natural killer T cells and γδ T cells are cytotoxic lymphocytes that overlap both innate and adaptive immunity. Cells from both arms of the immune system are in development as potential cellular immunotherapies.

The immune system is split into two arms: the innate and adaptive immune systems (Fig. 1). Through immune surveillance, any molecules that are identified as non-self are eliminated. Targets include not only virally infected cells but also transformed (tumour) cells, which can acquire antigenicity (see Box 1 for a glossary of terms) and hence immunogenicity through the expression of neo-antigens that can be recognised as non-self. However, cancer cells have developed strategies to escape and suppress the immune system (Pinzon-Charry et al., 2005; Blankenstein et al., 2012), which results in a failure to initiate and maintain adequate antitumour immunity, and consequently facilitates tumour survival and progression. Strategies include tumour antigens being only weakly immunogenic. Alternatively, the tumour might downregulate or modulate the expression of antigens, thus evading immune-cell detection. In addition, tumours can suppress the immune response through the synthesis of various immune suppressants that have roles in maintaining self-tolerance, or that inhibit effector immune cell function. Tumour immune suppression affects all branches of the immune system and can result in tumour escape from the immune system.

**Box 1. Glossary**

Allogeneic: derived from a different individual and hence genetically different from the host.
Anergy: a state of immune unresponsiveness. It is induced when the T cell’s antigen receptor is stimulated, effectively freezing T-cell responses pending a ‘second signal’ from the antigen-presenting cell.
Antigen-presenting cells (APCs): a heterogeneous group of cells that mediate a cellular immune response by processing and presenting antigens for recognition by T cells.
Antigenicity: the capacity of a molecule or an antigen to induce an immune response, i.e. to be recognised by and interact with an immunologically specific antibody or T cell receptor.
Autologous: derived from the same individual and hence genetically identical to the host.
Blast cells: these are the very earliest and most immature cells of the myeloid cell line.
Central tolerance: tolerance mechanisms that operate in the thymus before the maturation and circulation of T cells.
Dendritic cell (DC): a specialist antigen-presenting cell.
Effector CD8+ T cells: kill target cells expressing the cognate antigenic peptide target.
Graft-versus-host disease (GvHD): aggressive immune response caused when T cells derived from donor cells recognise the tissue of a recipient.
Human leukocyte antigen (HLA): highly polymorphic molecule required for antigen presentation encoded within the human major histocompatibility complex.
Leukapheresis: the selective separation and removal of white blood cells (leukocytes) from blood.
Major histocompatibility complex (MHC): proteins that control immune responses, encoded by a genetic locus encompassing a family of highly polymorphic genes.
Memory cell: a cell in the immune system that, when exposed to an antigen, replicates itself and remains in the lymph nodes searching for the same antigen, resulting in a more efficient and rapid response on repeat exposure (memory response).
Natural killer cell (NKC): a type of cytotoxic T cell that can be distinguished from a CD8^+^ T cell by its lack of TCR. They are part of the innate immune system.
Peripheral tolerance: not all self-antigens, which a T cell needs to be tolerant of, are expressed in the thymus. Peripheral tolerance is tolerance towards peripheral self-antigens that is developed after T cells mature and enter the periphery.
Regulatory T cells: a T-cell population that can functionally suppress the activity of other immune cells.
Senescence: loss of a cell’s power of division and growth.
Viral transduction: the transfer of genetic material to a cell via a viral vector.

As our understanding of the immune system has advanced, increasing numbers of innovative therapies are being developed that utilise the cells of the immune system and optimise their disease-targeted response through genetic modification. This is particularly true in the field of cancer medicine. By harnessing or enhancing the function of specific immune cells, the possibility exists to augment the immune response to achieve long-lasting cancer regression. Over the last 20 years, immune-cell therapies against cancer, based on the manipulation and infusion of autologous (derived from the same individual) or allogeneic (derived from other individuals) (see Box 1 for a glossary of terms) immune cells into patients, have been widely tested in clinical trials. These include natural killer cell (NKC; Box 1) therapies (Cheng et al., 2013; Miller, 2013), dendritic cell (DC; see Box 1) therapies (Palucka et al., 2011; Vacchelli et al., 2013) and genetically modified T-cell immunotherapies (Davila et al., 2014; **Maus et al., 2014a).

Given their increasing importance as a potential treatment for certain forms of cancer and their emerging clinical success, we focus this Review on the development of treatment strategies that use genetically modified T-cell immunotherapies. Advances in our knowledge of cancer immunology, improvements in the manufacturing of immunotherapy products and the ability to select patients whose tumours express specific antigens are improving the clinical outcomes of genetically modified T-cell immunotherapies. Nevertheless, and as we discuss, challenges remain in this emerging field related to the efficacy, safety and manufacturing of these therapies, as well as their regulation.

## T cells and the role of the T cell receptor

**Fig. 2.**
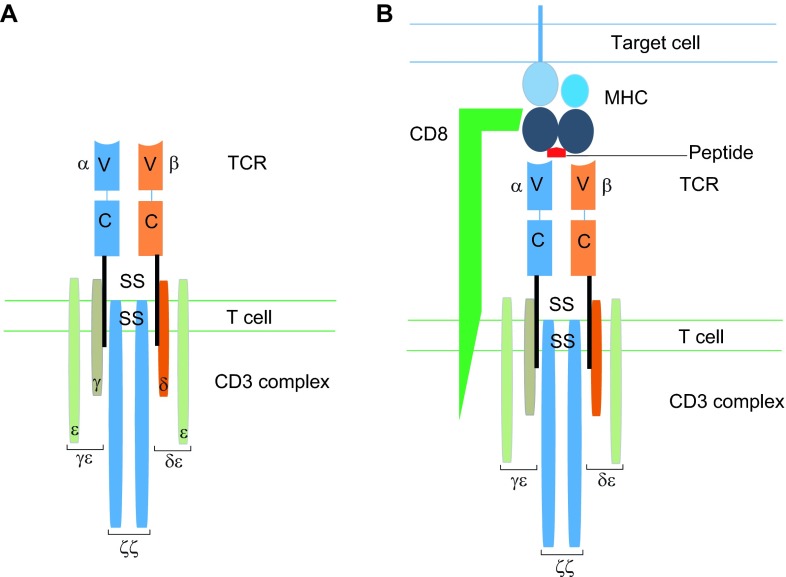
**Structure and function of the TCR.** (A) The T cell receptor (TCR), found on the surface of T cells, is responsible for antigen recognition. It consists of two chains: the alpha (α) and beta (β) chains. Both chains have a constant region (c) and a variable region (v), and it is the variable region that determines antigen specificity. The TCR is associated with the CD3 complex, which comprises three transmembrane signalling molecules (CD3ζζ, CD3δε and CD3γε). (B) A TCR will interact with an antigen on a target cell when the target peptide sequence is presented by the appropriate major histocompatibility complex (MHC-1 for cytotoxic T cells). Efficient T-cell activation also requires the simultaneous binding of the T cell co-receptor (CD8 for cytotoxic T cells). ss, disulphide bridge.

T cells (also known as T lymphocytes) are found widely distributed within tissues and the tumour environment. They play a central role in cell-mediated immunity and can mediate long-lived, antigen-specific, effector and immune memory responses. T cells are distinguished from other lymphocytes by the presence of T cell receptors (TCRs) on the cell surface. The TCR is a multi-subunit transmembrane complex that mediates the antigen-specific activation of T cells. The TCR is composed of two different polypeptide chains (Fig. 2A), the TCR α and β chains. Both chains have an N-terminal variable region and a constant region. The chains are linked by a disulphide bond, with each receptor providing a single antigen-binding site.

The TCR confers antigenic specificity on the T cell, by recognising an antigen ligand comprising a short contiguous amino acid sequence of a protein that is presented on the target cell by a major histocompatibility complex (MHC; Box 1) molecule (Fig. 2B). Accessory adhesion molecules expressed by T cells, such as CD4 for MHC class II and CD8 for MHC class I, are also involved. The TCR interacts with this ligand by making contacts with both the MHC molecule and the antigen peptide. Signal transduction is through the associated invariant CD3 complex, which is composed of four different CD3 proteins that form two heterodimers (CD3δε and CD3γε) and one homodimer (CD3ζζ) (Fig. 2A).

Following contact with their cognate peptides presented by MHC class I molecules, naive CD8^+^ cytotoxic T cells proliferate vigorously and acquire phenotypic and functional properties allowing them to act as effector T cells (Box 1); these eliminate cells expressing the antigen, through apoptosis-inducing ligands or release of lytic granules. In addition, long-lasting memory T cells (Box 1) are generated that can self-renew, allowing rapid expansion in the presence of the target antigen and providing a sustained and durable response to it upon re-exposure. The function of T cells as orchestrators and effectors of the adaptive immune response is directed by the specificity of the TCR.

## Central and peripheral tolerance

Although tumour antigens have the potential to be immunogenic, because tumours arise from the individual’s own cells only mutated proteins or proteins with altered translational processing will be seen as foreign by the immune system. Antigens that are upregulated or overexpressed (so called self-antigens) will not necessarily induce a functional immune response against the tumour: T cells expressing TCRs that are highly reactive to these antigens will have been negatively selected within the thymus in a process known as central tolerance (see Box 1) (Xing and Hogquist, 2012; Ruella and Kalos, 2014), meaning that only T cells with low-affinity TCRs for self-antigens remain.

The tumour environment also plays a key role in the immune response. For a T cell to become activated, co-stimulatory signals typically arising from antigen-presenting cells (such as dendritic cells; see Box 1) are required. However, tumour cells might insufficiently stimulate antigen-presenting cells, resulting in inadequate expression of MHC class I- and II-peptide molecules, co-stimulatory molecules and cytokine production (Hawiger et al., 2001). The antigen-presenting cells therefore cannot fully engage with the T cell. This leads to suboptimal T-cell activation, proliferation and expansion, resulting in anergy (peripheral tolerance; see Box 1). In addition, increasing evidence suggests that another cell type, regulatory T cells (T_Regs_; Box 1), have a principal role in suppressing tumour-specific T-cell activity and are a major barrier to immune responses against tumours (Ormandy et al., 2005; Zhou and Levitsky, 2007), leading to the establishment of an immune-suppressive state. The overall result is an increase in tumour survival; the goal of immune-cell-based therapies is to shift the balance of power back to the immune system.

## Genetically modified T cells in cancer immunotherapy

The concept of transferring T cells to patients (adoptive T-cell transfer) to treat disease has been established over many years through the *ex vivo* manipulation, expansion and reinfusion of T cells that target specific viruses, for example to treat viral infections, such as cytomegalovirus or Epstein Barr virus infections following haematopoietic stem cell transplantation (Walter et al., 1995; Heslop et al., 2010; Rooney and Leen, 2012). As described above, rare populations of tumour-antigen-specific T cells do exist and specifically can be isolated at the site of the tumour, and these are known as tumour infiltrating lymphocytes (TILs) (Kawakami et al., 1994; Robbins et al., 2013). TILs can be isolated from excised tumour tissue, cultivated, activated and expanded *ex vivo*, and, on reinfusion, have shown promising efficacy in the clinic, particularly in the treatment of melanoma (Rosenberg et al., 1988, Besser et al., 2013; Kvistborg et al., 2012; Dudley et al., 2013), supporting the therapeutic potential of tumour-specific T cells.

**Fig. 3.**
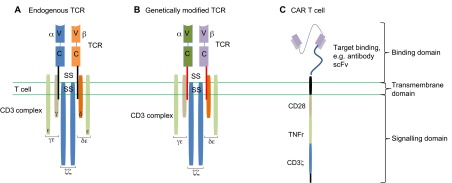
**Genetically modified TCRs for cancer immunotherapy.** (A) T-cell response can be manipulated and redirected against cancer, with improved specificity and affinity for tumour antigens, via genetic engineering of the endogenous TCR. (B) Genetically modified TCR: gene sequences are transferred to the T cell to encode new TCR α and β chains with different peptide specificity. In addition, there can also be transmembrane changes (red bars). To minimise interchain mispairing with the endogenous TCR, modifications such as the addition of a disulphide bridge (ss) are made. (C) Alternatively, a fusion receptor can be generated, a chimeric antigen receptor (CAR). Typically, these consist of three parts: a recognition sequence [represented here by an antibody-derived single-chain variable fragment (scFv)], a transmembrane element and an intracellular bespoke signalling domain (CD3ζ), which also contains co-stimulatory molecules, such as CD28 and tumour necrosis factor receptors (TNFr) such as OX-40.

An alternative option to these approaches that is now starting to generate compelling clinical data is based on the premise that the antigen specificity of T cells can be manipulated by genetic modification and redirected to successfully target antigens that are expressed by tumours. In particular, T cells can be engineered to express modified TCRs (so-called TCR therapies) or protein-fusion-derived chimeric antigen receptors (CARs) that have enhanced antigen specificity (Fig. 3). These approaches could overcome the fundamental limitations associated with central and peripheral tolerance, and generate T cells that will be more efficient at targeting tumours without the requirement for *de novo* T-cell activation in the patient.

### Genetically modified TCR therapies

Genetically modified TCR therapies are based on altering T-cell specificity through the expression of specific TCR α and β chains, which mediate the antigen-recognition process (Fig. 2). The tumour-specific TCR α and β chains are identified, isolated and cloned into transduction vectors and transduction of T cells creates tumour-antigen-specific T cells.

To generate a successful tumour-specific TCR, an appropriate target sequence needs to be identified. This might be isolated from a rare tumour-reactive T cell or, where this is not possible, alternative technologies can be employed to generate highly active anti-tumour T-cell antigens. One approach is to immunise transgenic mice that express the human leukocyte antigen (HLA; Box 1) system with human tumour proteins to generate T cells expressing TCRs against human antigens (Stanislawski et al., 2001). An alternative approach is allogeneic TCR gene transfer, in which tumour-specific T cells are isolated from a patient experiencing tumour remission and the reactive TCR sequences are transferred to T cells from another patient who shares the disease but is non-responsive (Gao et al., 2000; de Witte et al., 2006). Finally, *in vitro* technologies can be employed to alter the sequence of the TCR, enhancing their tumour-killing activity by increasing the strength of the interaction (avidity) of a weakly reactive tumour-specific TCR with target antigen (Robbins et al., 2008; Schmid et al., 2010).

### Chimeric antigen receptors (CARs)

CARs combine both antibody-like recognition with T-cell-activating function (Maher, 2012). They are composed of an antigen-binding region, typically derived from an antibody (Eshhar et al., 1993), a transmembrane domain to anchor the CAR to the T cell (Bridgeman et al., 2010), and one or more intracellular signalling domains that induce persistence, trafficking and effector functions in transduced T cells (Finney et al., 1998; Krause et al., 1998) (Fig. 3). Sequences used to define the antigen-targeting motif for a CAR are typically derived from a monoclonal antibody, but ligands (Muniappan et al., 2000) and other receptors (Zhang et al., 2012) can also be used.

CAR specificity is frequently determined by a single-chain variable fragment (scFv – the targeting domain), which is formed by the self-association of cloned variable regions of heavy and light chains of a monoclonal antibody (Fig. 3). The scFv is linked via a flexible spacer region to an intracellular signalling domain, typically the transmembrane and endodomain of the CD3ζ co-receptor. Co-stimulatory signals, such as those mediated by CD28, OX40 (a tumour necrosis factor receptor) and CD40L, enable a more efficient and long-lasting activation of T cells, but often tumours do not express appropriate ligands for such co-stimulatory molecules (Driessens et al., 2009). For this reason, although CARs that contain just CD3ζ (first-generation CARs) have been able to induce anti-tumour responses, in most cases the absence of co-stimulation has led to immune unresponsiveness (anergy) and to the failure of T-cell expansion *in vivo* (Heslop, 2010). Second-generation CARs, which include intracellular signalling domains for co-stimulatory signals such as CD28 and CD137, have been shown to produce enhanced tumour-regression effects (Carpenito et al., 2009). CARs that deliver more than one type of co-stimulatory molecule are now in development (Maher, 2012), as discussed further below.

CAR-expressing T cells (CAR T cells) recognise a variety of types of antigen, not only protein but also carbohydrate and glycolipid structures typically expressed on the cell surface of a tumour. Unlike for TCR recognition, the antigen does not need to be processed and presented by MHC and therefore the same CAR-based approach can be used in all patients who express the same tumour antigen regardless of HLA type.

## Efficacy and safety: lessons learned from clinical data

The first clinical trial that used genetically modified T cells for cancer therapy was a CAR-T-cell therapy and began in 1996 in patients with ovarian cancer (Kershaw et al., 2006). This and other early studies in a variety of cancers showed limited efficacy (Park et al., 2007; Till et al., 2008; Lamers et al., 2006; Kershaw et al., 2006). However, improvements in molecular biology and our understanding of immunology over the last two decades have now resulted in significant successes in clinical trial while clarifying some of the challenges remaining for the field.

### Clinical trials with TCR therapies

Initial clinical trials have demonstrated the overall feasibility and clinical potential of genetically modified TCR T cells (TCR therapies) as treatments for different types of cancer, with tumour regression being reported in patients (Table 1). The first clinical experience of such therapies was in individuals with melanoma. T cells were transduced with a TCR directed against the melanoma antigen recognized by T cells (MART1), which was cloned from a TIL isolated from a resected melanoma lesion. This TCR was specific for HLA-A2 individuals and was of low affinity for the target. Two out of 17 patients showed partial tumour regression, no significant toxicity and persistence of modified T cells for more than a year (Morgan et al., 2006). In addition to the original report, 31 patients were eventually treated in the trial (Lagisetty and Morgan, 2012); in total, four patients achieved measurable regression of metastatic melanoma. Although the number of responders was small, this was the first proof of principle for genetically modified TCR therapies. Other trials in this fast-growing field have subsequently demonstrated significant and prolonged tumour regression in patients with melanoma or sarcoma using genetically modified TCRs directed against MART1, melanoma-associated antigen 3 (MAGE-A3), glycoprotein 100 (gp100) and cancer testes antigen (NYESO-1) (Johnson et al., 2009; Robbins et al., 2011; Morgan et al., 2013; Morrison, 2014).

**Table 1.**
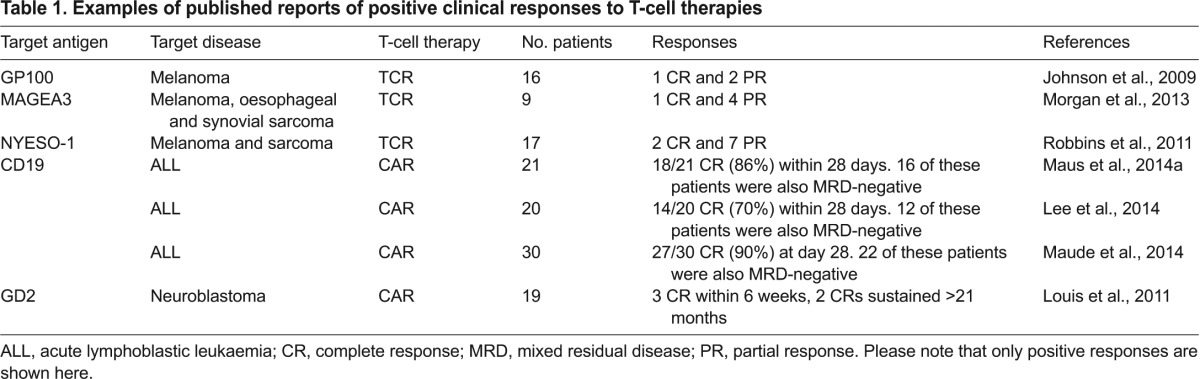
Examples of published reports of positive clinical responses to T-cell therapies

### Clinical trials with CAR T cells

The clinical evaluation of CAR therapies has grown exponentially, with the majority evaluating the treatment of B-cell cancers. Most B- cell malignancies as well as normal B cells express the CD19 antigen but this is absent from other cell types, making it an attractive therapeutic target. There are slight variations in the composition of the different anti-CD19 CAR T cells in trial (Maher, 2014) and the clinical trial designs have been variable (Kershaw et al., 2013), but several trials (Table 1) have now reported very impressive response rates in 60–90% of patients with relapsed or refractory lymphoblastic leukaemias (Maus et al., 2014a; Lee et al., 2014; Maude et al., 2014). Some responding patients have been consolidated with stem cell transplantation (Lee et al., 2014), whereas others have not, and sustained remissions of up to 2 years have been reported (Maude et al., 2014). It is currently unclear how long anti-CD19 CAR T-cell-induced remission can be sustained, but clearly this immunotherapy has the potential to be of significant clinical benefit. Following on from the great progress in B-cell malignancies, CAR T-cell therapies are also being developed that target solid tumours. This field is at an earlier stage although signals of efficacy have been observed in neuroblastoma (Pule et al., 2008; Louis et al., 2011).

### Factors that can affect efficacy

A number of factors are likely to contribute to the variability observed in clinical responses to genetically modified T-cell therapies. Persistence and survival of the genetically modified T cells is considered important and it has been reported that patients who have shown disappearance of all signs of cancer in response to treatment (complete responses) have also typically exhibited greater cell persistence and survival (Pule et al., 2008; Rosenberg et al., 2011). One factor that can impact the engraftment and persistence of transferred T cells is the use of preparative conditioning regimes (Klebanoff et al., 2005a; Dudley et al., 2008; Uttenthal et al., 2012). These regimes, commonly using fludarabine and/or cyclophosphamide, are administered to patients in order to reduce the number of circulating T cells (lymphodepletion). Lymphodepletion might promote the *in vivo* expansion of transferred cells by limiting the competition for cytokines such as interleukin (IL)-7 and IL-15, which promote proliferation of the existing T-cell compartment (Gattinoni et al., 2005b; Paulos et al., 2007). In addition, lymphodepletion will decrease the number of T_Reg_ cells, which, if present, could inhibit the anti-tumour activity of transferred genetically modified T cells (Schmitt et al., 2009).

Another factor that could influence the outcome of genetically modified T-cell therapy, but is hard to control for, is the cell dose. T-cell therapies are typically administered as a defined number of cells per kilogram of body weight; however, because T cells will replicate and expand after transfer, the administered cell dose does not resemble the final steady-state number of cells, which will vary among patients because the level of T-cell expansion will be patient-specific. Preclinical studies have shown a progressive improvement in tumour regression as the total number of adoptively transferred cells increased (Klebanoff et al., 2011). However, complete cancer remissions have been reported in patients with a range of administered anti-CD19 CAR T-cell doses, with anti-CD19 CAR T cells persisting for longer than 6 months (Kalos et al., 2011; Porter et al., 2011), indicating that the ability of the cells to proliferate and persist might be more important than the initial cell dose per se.

A factor that can influence the long-term maintenance of efficacy following genetically modified T-cell therapy is that cancer cells might downregulate or lose expression of the targeted antigens. Clinical trials of an anti-CD19 CAR T-cell therapy for the treatment of acute lymphoblastic leukaemia has suggested that this might occur (Grupp et al., 2013) and, recently, data has been reported on a group of patients that initially showed a complete response but who subsequently relapsed owing to the presence of blast cells (Box 1) that no longer expressed CD19 (Maude et al., 2014). The factors determining the risk of tumour-antigen loss remain to be elucidated.

Finally, many factors that are present in the tumour microenvironment can affect the efficacy of T-cell-based immunotherapies. The tumour microenvironment is composed of tumour cells, vasculature and immune cells, and is characterised by an immune-suppressive environment including lack of molecules that promote DC function, which affects antigen presentation with the potential to result in suboptimal T-cell activation and T-cell tolerance (Zou, 2005). In addition, tumours are characterised by the presence of large numbers of T_Reg_ cells, which reduce the cytolytic activity of tumour-specific T cells and also favour T-cell-tolerizing conditions. Tumours therefore propagate conditions that favour immune tolerance and this might impact the effectiveness of genetically modified T-cell therapies.

### Factors that can affect safety

Inevitably, there are potential safety risks associated with the use of genetically modified T-cell therapies (Table 2), with the most critical related to on-target off-tumour activity, off-target reactivity and cytokine-release syndromes (Casucci et al., 2015).

**Table 2.**
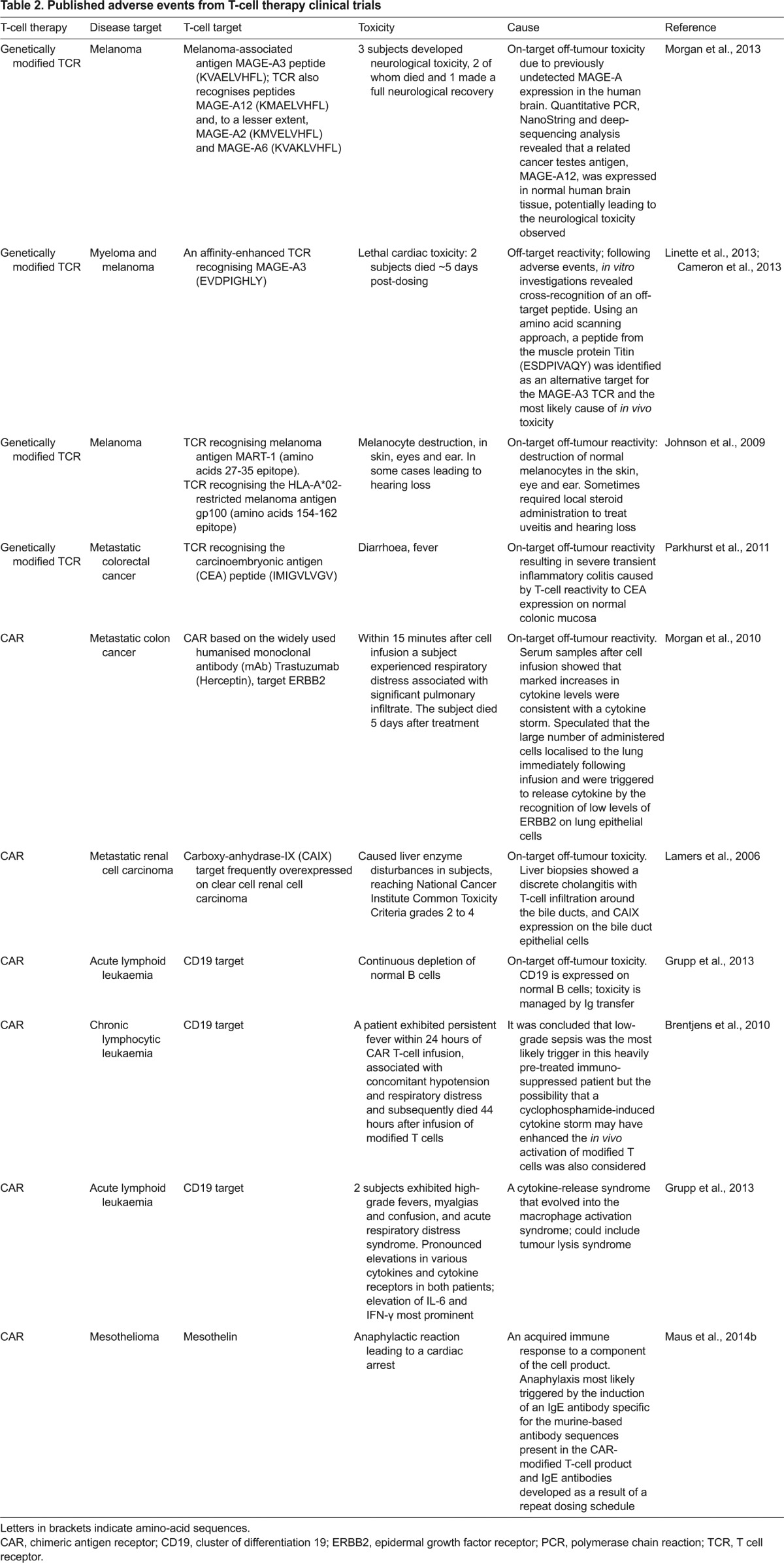
Published adverse events from T-cell therapy clinical trials

The availability and selection of an antigen target that is present only on tumour cells and not on normal cells is challenging and, where the antigen is expressed on normal cells, genetically modified T cells might trigger a potent cellular immune response against tissues of the body, even those that express the target antigens at low levels (Johnson et al., 2009). This type of reaction is known as on-target off-tumour activity (Casucci et al., 2015) and has been reported to occur in clinical trials (Table 2). For example, clinical trials involving anti-CD19 CAR T cells that have reported significant clinical efficacy have also reported that participants in these studies have shown continuous depletion of normal B cells, which also express CD19 (Grupp et al., 2013). Although in this case the on-target off-tumour toxicity can be managed by immunoglobulin transfer (to counteract the depletion of antibody-producing B cells), it highlights the challenges of identifying tumour-specific targets. On-target off-tumour toxicities are also a problem encountered with genetically modified TCR T-cell therapies (Linette et al., 2013). This is because the peptide target sequence of the TCR might also be present in other proteins (Cameron et al., 2013), making it important to screen extensively for potential targets of cross-reactivity. *In vitro* screening strategies are employed to reduce the risk that antigens targeted by a modified TCR are also present in vital organs (Cameron et al., 2013).

Several strategies are being explored to engineer T cells that have a higher selectivity for tumour than for normal tissue. These include dual-CAR targeting strategies (Lanitis et al., 2013; Pegram et al., 2014), in which T cells are modified to express simultaneously two CARs with different antigen specificities to ensure that T-cell activation only occurs on tumour cells, where both antigens are present (Kloss et al., 2013). Dual-CAR T cells show weak cytokine production against target cells expressing only one tumour-associated antigen, but demonstrate enhanced cytokine production upon encountering natural or engineered tumour cells expressing both antigens, and have also been reported to prevent tumour escape (Duong et al., 2011; Hegde et al., 2013). An alternative but more complex approach is the trans-signalling CAR strategy, whereby signal 1 of T-cell activation (mediated by antigen binding) is physically dissociated from the co-stimulatory signal 2 (usually mediated by CD28) in two CARs with different antigen specificity (Lanitis et al., 2013). These strategies demonstrate the principle that a dual approach might make genetically modified T-cell therapies safer.

**Fig. 4.**
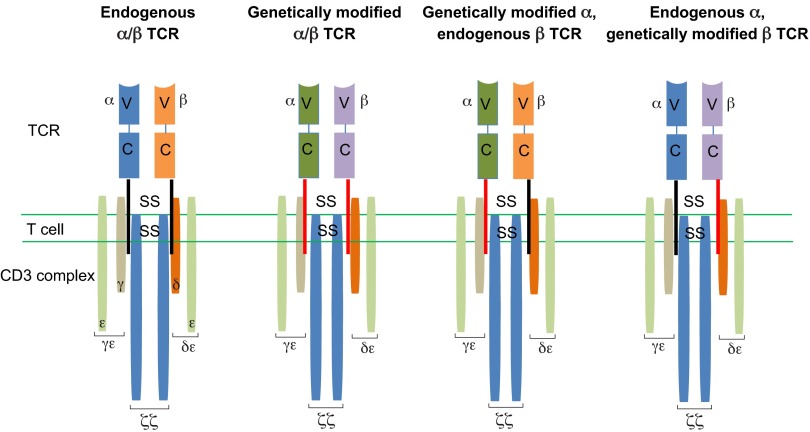
**TCR α- and β-chain pairing and mispairing.** A genetically modified TCR T cell expresses both the endogenous and transduced α/β TCR chains. There are four possible TCR chain combinations: (1) an endogenous α and β chain TCR; (2) a TCR generated from the transduced exogenous α and β TCR sequences; and (3 and 4) hybrid (mispaired) TCRs formed from a combination of endogenous and exogenous α and β chains. The reactivity of the hybrid TCRs is unknown and is a potential source of self-reactive toxicities.

Another undesirable reaction that can occur is off-target reactivity and has also been reported in clinical trials (Table 2). This can occur as cross-reactivity and is particularly a risk for genetically modified TCR T cells, which could react against peptides in proteins other than the targeted ones. Other causes of off-target toxicity include generation of unpredicted specificities through TCR mispairing (described in more detail below) between endogenous and introduced α/β TCR chains (Fig. 4). Although in clinical trials to date no formal observations of toxicities mediated by TCR mispairing have been observed, preclinical studies have clearly demonstrated that TCR mispairing has the potential to induce harmful recognition of self-antigens, resulting in graft versus host disease (see Box 1 for a glossary of terms) (Bendle et al., 2010; van Loenen et al., 2010).

Cytokine-release syndrome has been reported following treatment with genetically modified T-cell therapies (Maus et al., 2014a). Recent clinical data have shown that the T-cell therapies can be very effective against the target tumour by inducing tumour cell lysis and potentially tumour cell removal at a faster rate than is seen with traditional immune therapies. This can result in high levels of cytokine release and macrophage activation syndrome, and subjects in clinical trials have exhibited profound cytokine symptoms such as very high fevers, rigors, nausea and diarrhoea (Table 2) associated with high levels of IFN-γ and a significant increase in IL-6 (Maude et al., 2014). Studies have shown (Teachey et al., 2013) that administration of anti-IL-6 receptor antibody can inhibit these reactions. Studies are ongoing to investigate the optimal timing of anti-IL6 treatment and it is not yet known whether this impacts efficacy of the therapies. In a clinical context, at least some level of cytokine release syndrome might be an inevitable consequence of efficacy of these therapies.

## New strategies for improving current T-cell-based immunotherapies

In order to build on and broaden the early clinical successes of genetically modified therapies, there are three key challenges that need to be addressed: appropriately activating T cells upon antigen recognition; counteracting the immunosuppressive effects of the tumour environment; and identifying new tumour-specific antigens (Kunert et al., 2013).

### T-cell activation

The functional activation and proliferation of T cells is determined not only by interactions between the T cell and its target but also by T-cell co-stimulatory signals. Tumours, however, often present antigens in the absence of co-stimulatory ligands, which can result in exhausted T cells with reduced proliferative capacity and effector function (Capece et al., 2012). Alternatively, the tumour environment might induce an upregulation of T-cell co-inhibitory molecules, which compromise tumour-specific T-cell responses (Norde et al., 2012). Both TCRs and CARs are being developed with a signalling cassette that harbours a co-stimulatory molecule that should provide a stimulatory trigger to the T cell even when one is not provided by tumour cells (Schaft et al., 2006; Maher, 2012). Alternatively, prior to transfer into patients, T cells can be stimulated *ex vivo* with human artificial antigen-presenting cells that express co-stimulatory ligands (Singh et al., 2011); this process has the potential to improve function *in vivo*.

Another factor important in maximising the activation of genetically modified TCR T-cell therapies is to minimise the formation of mixed TCR dimers (TCR mispairing) between the genetically modified TCR and the endogenous TCR expressed by the patient’s T cells. There are theoretically four possible TCR specificities (Fig. 4) between the native and genetically modified TCR. TCR mispairing could significantly decrease the functional avidity of the genetically modified T cells by reducing the ability of the cells to interact with the desired target peptide and, in addition, it can potentially represent a risk for autoimmunity.

Numerous strategies have been employed to minimise the risk of mispairing (Govers et al., 2010). One option is to use murinised TCRs. It has been shown that murine TCRs are more efficiently expressed in human T cells than human TCRs (Cohen et al., 2006). Human TCRs that have the constant domains replaced with murine sequences preferentially bind with each other rather than the endogenous TCR (Cohen et al., 2006; Thomas et al., 2007; Sommermeyer and Uckert, 2010). An alternative option is to introduce a new intramolecular disulphide bond into the extracellular α and β chain C-terminus domains through additional cysteine residues (Thomas et al., 2007; Kuball et al., 2007; Cohen et al., 2007). Alternatively, the insertion of point mutations into the α and β chain C domains improves specific pairing for the introduced TCR (Voss et al., 2008; Haga-Friedman et al., 2012). Recently, an alternative strategy has been explored that attempts to limit TCR mispairing by removing or reducing endogenous TCR chain expression (Provasi et al., 2012; Bunse et al., 2014).

**Fig. 5.**
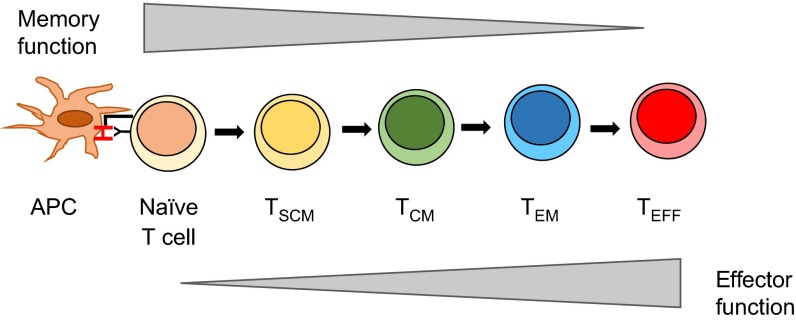
**CD8^+^ T-cell subsets.** There are a number of different CD8^+^ T-cell subsets. Naïve, T stem cell (T_SCM_) and T central memory (T_CM_) cells circulate and migrate to lymphoid tissue, whereas effector memory T cells (T_EM_) and effector T cells (T_EFF_) have the capacity to traffic to peripheral tissues. There are a number of models for the differentiation of CD8^+^ T cells (Joshi and Kaech, 2008). One model is the linear model for differentiation of CD8^+^ T cells, which proposes that, following activation of a naïve T cell, there is a progressive differentiation through three major circulating subsets of T cells (T_SCM_, T_CM_ and T_EM_), with T_EFF_ representing the terminally differentiated T cells. Targeting different T-cell subsets could increase efficacy and persistence of genetically modified T-cell therapies.

Finally, strategies to manipulate T-cell differentiation in favour of specific T-cell types that might better counteract tumour cells are also being considered. The effector and memory functions of CD8^+^ T cells are implemented by functionally distinct subsets (Fig. 5). By exposing T cells to γ-cytokines such as IL-7 and IL-15 (Kaneko et al., 2009) or IL-15 and IL-21 (Pouw et al., 2010) prior to adoptive T-cell transfer to drive T-cell differentiation, gene-engineered T cells that have a central memory phenotype, prolonged peripheral persistence and potent antigen reactivity have been generated (Kaneko et al., 2009; Pouw et al., 2010). An alternative approach is focused on the direct selection, isolation and transfer of specific genetically modified CD8^+^ T-cell populations (Hinrichs et al., 2009; Uttenthal et al., 2012). However, data are accumulating that a combined CD4^+^ and CD8^+^ T-cell response might provide a therapeutic advantage and that selecting single-cell populations might risk limiting therapeutic efficacy (Moeller et al., 2005; Moeller et al., 2007).

### Countering the immunosuppressive effects of the tumour environment

Current research into the development of improved genetically modified T-cell therapies is also focused on strategies to optimise the tumour microenvironment and address the imbalance between the number and activation state of immune effector T cells versus those of suppressor cells (such as T_Regs_). Active research areas to counteract this imbalance include investigating the role of molecules that are involved in effector-T-cell migration into tumour tissues as part of patient conditioning, and the beneficial use of co-treatments, such as chemotherapy and cytokine support (Pegram et al., 2012). Strategies that remove or deplete immune-suppressor cells in combination with adoptive T-cell therapy might enhance anti-tumour responses in cancer immunotherapies. A further strategy receiving attention is the potential to combine gene-engineered T-cell therapy with the newly available anti-checkpoint antibodies [anti cytotoxic T-lymphocyte-associated protein 4 (CTLA4) or anti programmed cell death protein 1 (PD1)] (John et al., 2013). Checkpoint inhibitors act as immunological ‘checkpoints/brakes’ preventing overactivation of the immune system on healthy cells. Tumour cells utilise these checkpoints to escape detection by the immune system. Combinations of genetically modified T-cell therapies and anti-checkpoint inhibitor therapies could have an important role in immunotherapy of solid tumours.

Additional approaches for enhancing the activity of the introduced T cells in the tumour environment include: modifying the introduced T cells to secrete IL-12, which promotes intrinsic resistance to T_Reg_-cell-mediated inhibition (Pegram et al., 2012); generating modifications to enhance T-cell trafficking and infiltration into cancer tissues through co-expression of chemokines (Kershaw et al., 2013); improving T-cell survival through provision of cytokine support (Kershaw et al., 2013); and investigating the delivery of genetically modified receptors to more immature T-cell populations [e.g. T stem cell memory (T_SCM_) cells], because such cells might exhibit less effector function but have greater capacity for *in vivo* survival and proliferation (Fig. 5) (Gattinoni et al., 2011).

### Identifying new tumour antigens

The differences between normal and cancer cells are in many cases subtle. Molecules that are tumour-specific or overexpressed in cancer are likely to have functional roles that participate in cellular transformation and migration. Deregulation of signal transduction pathways in cancer pathogenesis, for example, is well established (Jones et al., 2008) and protein phosphorylation is the dominant process (Hanahan and Weinberg, 2011). Recently, research has shown that tumours can produce tumour-specific phosphopeptides and that healthy individuals display immune responses with memory characteristics against many of them, suggesting that they might be important targets for immunotherapies (Cobbold et al., 2013). Cancer cells also frequently alter the glycoproteins they display, either through increased production or increased branching on the glycan structures (Fuster and Esko, 2005). Differences in these structures might be sufficient to allow tumour-specific targeting. Cancer cells also produce mutated epitopes and these can be recognised by T cells (Lu et al., 2013; Robbins et al., 2013; Lu et al., 2014), and CAR T-cell therapies targeting mutated targets are in clinical trials.

## Manufacturing and regulatory challenges of T-cell-based therapies

**Fig. 6.**
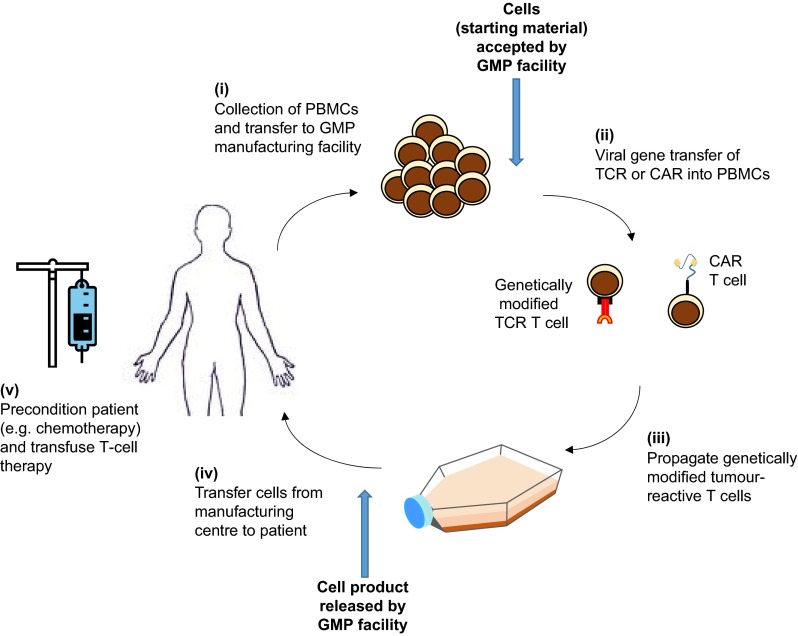
**Manufacturing and delivery pipeline of genetically modified T-cell therapies.** (i) T cells are harvested from a patient and sent to a good manufacturing practices (GMP) manufacturing facility, which might not be local to the treating hospital. Cells that pass acceptance criteria are genetically engineered (ii) with either a new T cell receptor (TCR) or a receptor based on a recognition sequence of an antibody [chimeric antigen receptor (CAR)], combined with T-cell co-stimulatory sequences. After a brief period of *in vitro* expansion and passing of product-specific release criteria (iii), the T-cell product must be returned to the correct patient (iv). The patient can undergo conditioning regimens prior to infusion of the genetically modified T-cell product (v). The complexity of this multi-step process in the manufacture and delivery of T-cell immunotherapies poses several economic and regulatory issues, which represent a challenge for the improvement and accessibility of such therapies. PBMC, peripheral blood mononuclear cell.

To date, the cost and complexity associated with the manufacture of personalised genetically modified T-cell therapies (Fig. 6) has restricted their production and use to specialised centres treating relatively small numbers of patients. The advances in clinical data highlighted above are attracting increasing commercial interest, including from large pharmaceutical companies (Flemming, 2014), and this investment is needed because these therapies will only become the standard of care if the cost, volume and regulatory challenges associated with their manufacture are addressed. Ultimately, these therapies might become available from a scalable allogeneic ‘off the shelf’ source (Gouble et al., 2014), but currently, and based on the compelling clinical need, the manufacturing of patient-specific therapies must be addressed.

### Manufacturing challenges

The manufacturing process must consistently successfully produce the final product to the required specification, but this is challenging given the variability within the starting material (either whole blood or leukapheresis material; see Box 1). There is inherent inter-patient variability in terms of numbers of cells and cell subsets due to natural variation, disease status and previous treatments received by the patient. Protocols are therefore needed to optimise the collection, purification and activation of T-cell products. Additionally, as trials progress and products enter clinical use, storage and stability of the starting material and final product will need to be addressed, including the ability to cryopreserve the product. Another major challenge is the logistics, with the tracking of patient-specific material from patient to and through the manufacturing centre and back again.

A critical manufacturing step is T-cell transduction with the viral vector (typically gamma retrovirus or lentivirus; Box 1) to introduce the genetic modification (Fig. 6ii). The growth in the field of genetically modified T-cell immunotherapy has resulted in the need for extra manufacturing capability and capacity for viral vectors. Improvements both in viral vectors and transduction methods to increase transduction efficiency, hence improving yield and decreasing the use of expensive vectors, are important and recent advances have been made (Casati et al., 2013; Dodo et al., 2014).

T-cell therapies undergo an expansion phase during manufacture to increase T-cell number (Fig. 6iii). A balance is required between maximizing T-cell number and the time required to achieve this, without the concomitant risks of T-cell senescence (Box 1) (Gattinoni et al., 2005a; Tran et al., 2008) and delay in product availability for fragile patients. Studies have indicated that T-cell survival and proliferation *in vivo* might be dependent on the differentiation status of the infused T cells (Berger et al., 2008; Hinrichs et al., 2009; Hinrichs et al., 2011; Klebanoff et al., 2011). Protocols in which less-differentiated T-cell subsets have been expanded, for example through substitution of IL-15 for IL-2 during manufacture, have exhibited greater proliferative capacity and persistence in nonclinical and clinical studies (Robbins et al., 2004; **Gattinoni et al., 2005b; **Klebanoff et al., 2005b; Montes et al., 2005; Klebanoff et al., 2012). This might therefore improve efficacy and this remains an area of on-going research.

### Regulatory environment

Genetically modified T-cell therapies are regulated under the Advanced Therapy Medicinal Product Regulation in the EU, within the Center for Biologics Evaluation and Research (CBER) Office of Cellular, Tissue and Gene Therapies at the FDA in the US, and under the new Pharmaceuticals and Medical Devices Law in Japan. It is recognised that the field is advancing very quickly both in terms of the science and emerging compelling clinical data. Advancements in the regulation are therefore needed and are taking place to respond to the scientific and clinical progress. These factors, combined with the preponderance of academic groups in this field, underline the importance of seeking regulatory interactions and guidance from an early stage of development.

In the preclinical area, regulatory guidance is available on the requirements for preclinical testing (Table 3). For genetically modified T cells, in addition to evaluating the safety of the product and the viral vector involved, the challenge is to evaluate the relevance and utility of efficacy models. In the majority of cases, utilisation of a risk-based approach will help the development of the nonclinical strategy (Table 3).

**Table 3.**
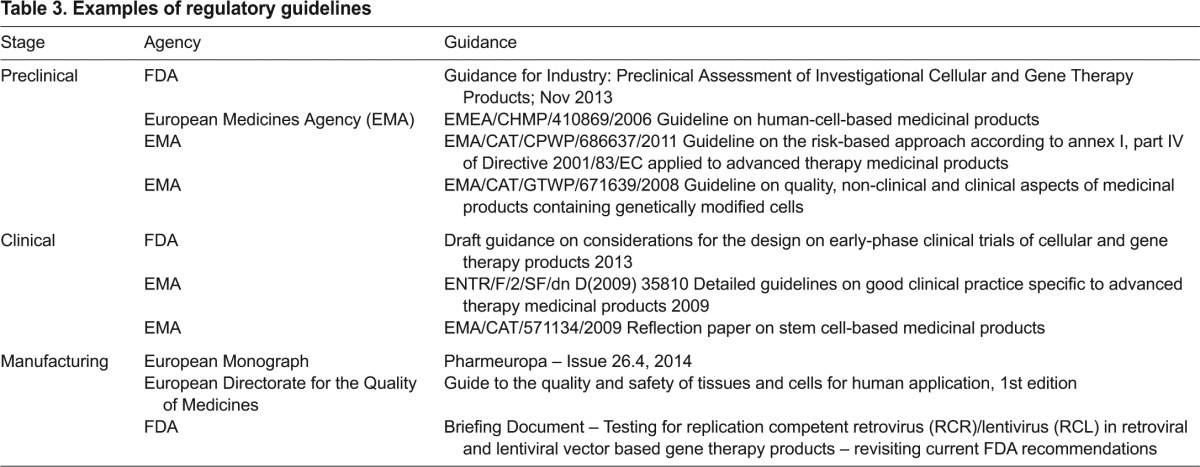
Examples of regulatory guidelines

Clinically, trial design in this area does not proceed along the traditional Phase I-II-III development pathway but starts directly in patients and often proceeds in a more seamless manner to collect the data required. Trial design, including comparators (controlled trials with an established effective treatment) and randomisation, can be challenging along with the requirements for long-term follow-up of patients who have received genetically modified therapies. It is likely that patient registries will be needed in order to fulfil long-term follow-up needs. Points to consider in this area are also available from the regulators (Table 3).

Meeting regulatory requirements for the manufacturing process means addressing the technical challenges described previously. These include setting suitable specification to allow for patient variability both in starting material and final product as well as conducting suitably extensive final product characterisation to enable comparison of results within trials and following any changes in the product manufacture process or manufacturing site. During the development process and to reach the scale required, further manufacturing optimisation will also be needed to include the use of fully good manufacturing practices (GMP) reagents and ideally remove serum-containing steps. It is recognised that GMP quality reagents are not always available and raw materials of biological origin are often required; a draft European monograph on this subject has recently been published (Table 3).

Another area of recent focus is the ongoing requirement in some jurisdictions for extensive and repeated viral-vector replication-competency testing. This stemmed from early trials but recent experience with more modern vector constructs have shown no evidence of replication competency in vectors designed to be replication incompetent, with no positive replication competency results in samples of retrovirus or lentivirus vector lots that were used for clinical studies in the past 10 years (Table 3). It therefore seems reasonable to suggest that the extent of replication-competency testing currently required in some jurisdictions could be reduced.

If autologous therapies are to become widely available to patients in the long term then the regulatory licensing framework for manufacturing would also ideally be able to accommodate an appropriate model for the regulation of hospital sites involved in point of care or final-stage manufacturing steps. Under the current EU framework, for example, such sites are currently required to hold a full manufacturing licence for these activities. An alternative model could be envisaged whereby these sites become satellite manufacturing sites under a main licence holder under appropriate quality oversight.

Finally, expediting regulatory approvals to allow patients in different regulatory jurisdictions to access innovative therapies is crucial. Recently, there has been innovation in models to speed progress through the regulatory path or access for patients in the US (Sherman et al., 2013), in Japan [Pharmaceuticals and Medical Devices Law with provisions for Regenerative Medicine (Hara et al., 2014)], the EU [adaptive licensing pilot (Eichler et al., 2012)] and the UK [the Medicines and Healthcare Products Regulatory Agency (MHRA) early access to medicines scheme]. These schemes are welcomed as promising clinical data emerges.

Patient access will also depend on the commercialisation models for a single personalised product. Current quoted costs in the literature are around £25,000 per product (Kunert et al., 2013; Buckland and Gaspar, 2014). However, these costs relate only to cell production. The true cost of a therapy must also consider medical costs such as inclusion of lymphodepleting preparative regimens, length of hospitalisation and co-administration of other agents with the cells (Weber et al., 2011). This will require the development of clear models of cost versus benefit.

## Conclusions

The feasibility of T-cell adoptive transfer was first reported nearly 20 years ago (Walter et al., 1995) and the field of T-cell therapies is now poised for significant clinical advances. Recent clinical trial successes have been achieved through multiple small advances, improved understanding of immunology and emerging technologies. As the key challenges of T-cell avidity, persistence and ability to exert the desired anti-tumour effects as well as the identification of new target antigens are addressed, a broader clinical application of these therapies could be achieved. As the clinical data emerges, the challenge of making these therapies available to patients shifts to implementing robust, scalable and cost-effective manufacture and to the further evolution of the regulatory requirements to ensure an appropriate but proportionate system that is adapted to the characteristics of these innovative new medicines.
